# Cirsiliol and Quercetin Inhibit ATP Synthesis and Decrease the Energy Balance in Methicillin-Resistant *Staphylococcus aureus* (MRSA) and Methicillin-Resistant *Staphylococcus epidermidis* (MRSE) Strains Isolated from Patients

**DOI:** 10.3390/molecules28176183

**Published:** 2023-08-22

**Authors:** Silvia Ravera, Gabriele Tancreda, Luigi Vezzulli, Anna Maria Schito, Isabella Panfoli

**Affiliations:** 1 Department of Experimental Medicine, University of Genoa, 16132 Genoa, Italy; silvia.ravera@unige.it (S.R.);; 2 Department of Earth, Environmental and Life Sciences (DISTAV), University of Genoa, Corso Europa 26, 16132 Genoa, Italy; 3 Department of Surgical Sciences and Integrated Diagnostics (DISC), University of Genoa, 16132 Genoa, Italy; 4 Department of Pharmacy (DIFAR), University of Genoa, 16132 Genoa, Italy

**Keywords:** ATP synthesis, F_1_F_o_-ATP synthase, flavonoids, methicillin-resistant *Staphylococcus aureus* (MRSA), methicillin-resistant *Staphylococcus epidermidis* (MRSE), cirsiliol, quercetin, oligomycin

## Abstract

Polyphenols have attracted attention in the fight against antibiotic-resistant bacteria, as they show antibacterial action. Considering that polyphenols inhibit F_1_F_o_-ATP synthase (ATP synthase) and that bacteria need a constant energy production to maintain their homeostasis, we evaluated the effect of two flavones, cirsiliol (tri-hy-droxy-6,7-dimethoxyflavone) and quercetin (3,3,4,5,7-pentahydroxyflavone), on energy production and intracellular ATP content in a methicillin-resistant *Staphylococcus aureus* (MRSA) strain and a methicillin-resistant *Staphylococcus epidermidis* (MRSE) strain isolated from patients, comparing the results to those obtained by treating the bacteria with oligomycin, a specific ATP synthase F_o_ moiety inhibitor. Real-time quantitative ATP synthesis and total ATP content of permeabilized Gram-positive bacteria were assayed by luminometry. The results showed that cirsiliol and quercetin inhibited ATP synthase and decreased the intracellular ATP levels in both strains, although the effect was higher in MRSE. In addition, while cirsiliol and quercetin acted immediately after the treatment, oligomycin inhibited ATP synthesis only after 30 min of incubation, suggesting that the different responses may depend on the different permeability of the bacterial wall to the three molecules. Thus, cirsiliol and quercetin could be considered potential additions to antibiotics due to their ability to target ATP synthase, against which bacteria cannot develop resistance.

## 1. Introduction

Polyphenols are a class of natural compounds that exert pleiotropic effects on various aspects of cell biology [1,2], and among them, flavonoids are receiving particular attention for their therapeutic potential [3,4,5]. Plants synthesize flavonoids as a defense mechanism in response to pro-oxidant insults [6], and, as far as human health is concerned, the intake of flavonoids from plants and fruits plays a pivotal role in the prevention and modulation of a wide range of chronic diseases [5]. Structurally, flavonoids are characterized by three bound rings that differ in saturation and hydroxylation patterns, distinguishing the various subclasses of flavonoids (flavonols, flavones, flavanones, flavanonols, isoflavones, aurones, anthocyanins, and chalcones) [7]. Flavonoids act mainly as antioxidants as they inhibit several pro-oxidant pathways linked to aging [8,9,10,11] or because they have a metabolism-modulating action [12,13]. Among the most extensively studied flavonoids, quercetin (3,3,4,5,7-pentahydroxyflavone) is widely distributed in the plant kingdom and a common ingredient in the human diet [14]. Quercetin possesses antioxidant and anti-aging properties due to its ability to donate hydrogen atoms, eliminate reactive oxygen species, inhibit xanthine oxidase [15,16]. The antioxidant effects of quercetin as well as its high bioavailability play a significant role in the prevention and treatment of some chronic diseases, such as cancer and cardiovascular diseases [15,16]. In recent years, among flavones, cirsiliol (6,7-dimethoxy-5,3′,4′-trihydroxyflavone) has also assumed a relevant role in human health as it has been shown to possess anti-inflammatory [17], antitumor [18,19], and sedative–hypnotic activity [20]. In detail, cirsiliol (6,7-dimethoxy-5,3′,4′-trihydroxyflavone) is present in several plants belonging to the family Lamiaceae, which includes about 200 genera, such as *Salvia* spp. [21], the aerial parts of the plant *Isodon rubescens* (also known as *Rabdosia rubescens* (Hemsl.) [22], and *Leonotis nepetifolia* (Lamiaceae) [23]. On the other hand, extracts of these plants are used in herbal medicine for inflammatory disease treatment due to their free-radical scavenging action [24,25]. Besides the scavenging effect, quercetin and cirsiliol act as antioxidant molecules as they inhibit the F_1_F_o_ ATP synthase (ATP synthase) [21,26,27], the nanomachine belonging to the oxidative phosphorylation responsible for ATP production. Indeed, quercetin and cirsiliol block the rotation of the gamma subunit ATP synthase by binding to a common site on the inner surface of the N-terminal domains of the α and β subunits of the F_1_ moiety [28]. This inhibiting action results in a slowing down of the electron transport chain, coupled to the ATP synthase, and at the same time of the production of reactive oxygen species, as demonstrated in the rod outer segments of the bovine retina [21,26]. Comparison of the major binding modes of cirsiliol and cisquercetin with the F_1_ moiety of bovine ATP synthase by molecular docking analyses showed that the chains involved in the binding are the same, as are the interacting amino acids, with some of them (Ser-267.G), specific to cirsiliol binding, allowing the latter to bind the enzyme with a higher binding energy [21]. On the other hand, the inhibiting action on ATP synthase is common to other polyphenols such as piceatannol and resveratrol [28]. However, flavonoids also act as broad-spectrum antibacterial agents [29,30], and this effect may be also due to the reduction in ATP synthase activity [28,31], as bacteria require constant energy production for the management of protein synthesis and DNA replication to maintain their high proliferation rate [32].

Thus, this work aimed to evaluate the effects of flavonoids on the ATP synthase of bacteria with clinical relevance. In detail, we treated one strain of methicillin-resistant *S. aureus* (MRSA) and one strain of methicillin-resistant *S. epidermidis* (MRSE) with cirsiliol and quercetin, comparing the results with data obtained in the presence of oligomycin, a specific ATP synthase inhibitor. We chose these strains because MRSA and MRSE pose a major threat to human health worldwide, as over the years, they have developed resistance to antibiotics [33,34,35,36]. In addition, based on our knowledge, this manuscript represents one of few protocols to measure the time course of ATP synthesis in bacteria, as only another method is available in the literature for the quantitative measurement of Gram-negative bacterial ATP synthesis [37].

## 2. Results

### 2.1. Evaluation of Aerobic ATP Synthesis in MRSA_A and MRSE_178

**Figure 1:**
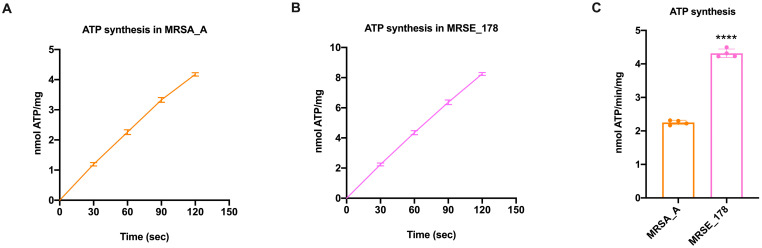
**Aerobic ATP synthesis evaluation in *MRSA strain A and MRSE strain 178***. (**A**) ATP synthesis time course in *MRSA strain A (MRSA_A)*. (**B**) ATP synthesis time course in *MRSE strain 178 (MRSE_178)*. (**C**) Comparison of ATP synthesis in *MRSA_A* and *MRSE_178*. In each panel, the data are presented as mean ± SD and are representative of four independent experiments. In panel C, **** indicates *p* < 0.0001 between *MRSA_A and MRSE_178*, evaluated by Student’s *t*-test.

Since, according to the endosymbiont theory, mitochondria are derived from bacteria [38], we evaluated the aerobic ATP synthesis in MRSA strain A (MRSA_A) and MRSE strain 178 (MRSE_178). To the best of our knowledge, aerobic ATP synthesis evaluation in bacteria is very complex due to the presence of a thick cell wall, which hinders both the entry of the substrates used for the assay (thereby including ADP) and the exit of newly formed ATP, so that it can be detected by the luciferin–luciferase system (see Materials and Methods). By modifying the method proposed by Hara and Mori for *E. coli* [37], so to adapt it to Gram-positive bacteria, we observed that MRSA_A and MRSE_178 showed a similar time course of ATP synthesis (Figure 1A,B). However, when comparing the ATP quantity produced in the same time unit, MRSE_178 displayed a three-fold higher production than MRSA_A (Figure 1C).

### 2.2. ATP Synthesis in MRSA_A and MRSE_178 Is Sensitive to Flavonoids but Apparently Not to Oligomycin

**Figure 2:**
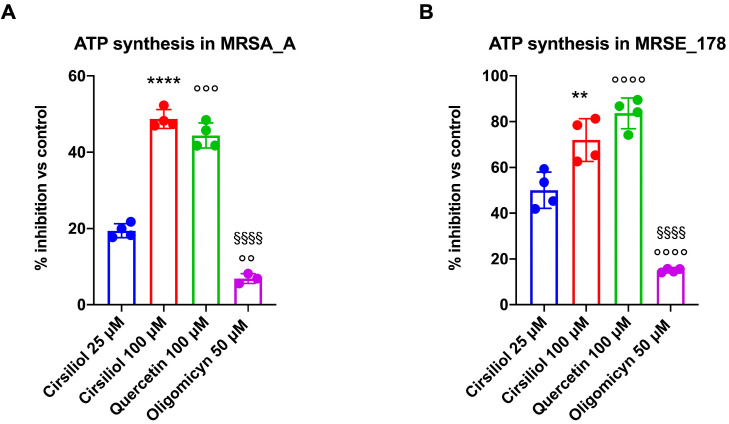
**Cirsiliol, quercetin, and oligomycin effects on ATP synthesis in MRSA strain A and MRSE strain 178.** (**A**) Percentage of ATP synthesis inhibition in MRSA strain A (MRSA_A) following treatment with cirsiliol (25 and 100 μM), quercetin (100 μM), and oligomycin (50 μM). (**B**) Percentage of ATP synthesis inhibition in MRSE strain 178 (MRSE_178) following treatment with cirsiliol (25 and 100 μM), quercetin (100 μM), and oligomycin (50 μM). In each panel, data are presented as mean ± SD and are representative of four independent experiments. The difference significance was tested by a one-way ANOVA test followed by the Tuckey’s test. ** and **** indicate *p* < 0.01 or 0.0001 between 25 μM or 100 μM cirsiliol; °° and °°° and °°°° indicate *p* < 0.01 or 0.001 or 0.0001 between 25 μM cirsiliol and quercetin or oligomycin treatments; §§§§ indicate *p* < 0.0001 between 100 μM cirsiliol and quercetin or oligomycin treatments.

Literature data show that several flavonoid compounds inhibit ATP synthase in eukaryotic cells, slowing oxidative phosphorylation and reducing ATP production [26,28,39,40]. We, therefore, wondered whether these compounds could also have the same effect on ATP synthase in bacteria, representing a possible strategy to reduce the intracellular ATP pool. In particular, our attention focused on cirsiliol, a dimethoxyflavone capable of binding to the F_1_ moiety of ATP synthase by inhibiting its rotation [21], comparing the data to those obtained after treatment with quercetin, a pentahydroxyflavone with similar action to cirsiliol [28], and oligomycin, a specific inhibitor of the F_o_ moiety of ATP synthase [41]. The data showed that cirsiliol inhibited ATP synthesis in both MRSA_A and MRSE_178 in a dose-dependent manner and that the highest cirsiliol concentration tested exerted an effect similar to that achieved with the same quercetin concentration (Figure 2A,B). However, cirsiliol and quercetin showed a higher inhibitory effect in MRSE_178 than in MRSA_A. In contrast, oligomycin displayed a slight inhibitor effect on ATP synthase in both strains, despite being considered this enzyme’s most specific inhibitor (Figure 2A,B).

### 2.3. Oligomycin Exerts Its Inhibitor Effect on ATP Synthase after a Half Hour of Incubation before the ATP Synthesis Assay

**Figure 3:**
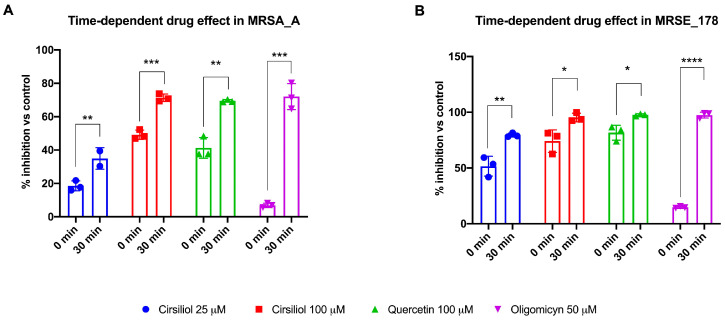
**Cirsiliol, quercetin, and oligomycin effects on ATP synthesis in MRSA strain A and MRSE strain 178 after 30 min of incubation**. (**A**) Percentage of ATP synthesis inhibition in MRSA strain A (MRSA_A) following treatment with cirsiliol (25 and 100 μM), quercetin (100 μM), and oligomycin (50 μM) before or after 30 min of drug incubation. (**B**) Percentage of ATP synthesis inhibition in MRSE strain 178 (MRSE_178) following treatment with cirsiliol (25 and 100 μM), quercetin (100 μM), and oligomycin (50 μM) before or after 30 min of drug incubation. In each panel, data are presented as mean ± SD and are representative of three independent experiments. The difference significance was tested by a one-way ANOVA test followed by the Tuckey’s test. *, **, ***, and **** indicate *p* < 0.05, 0.01, 0.001, or 0.0001 between the samples incubated or not with the drugs for 30 min.

To understand whether the inability of oligomycin to inhibit bacterial ATP synthesis could depend on a slow internalization of the molecule, MRSA_A and MRSE_178 were incubated for half an hour with cirsiliol, quercetin, or oligomycin before the ATP synthesis assay. The data showed that the inhibition of MRSA_A and MRSE_178 ATP synthetic activity by cirsiliol and quercetin, which was already evident without pre-incubation, increased even further, reaching approximately 75% for both flavonoids (Figure 3A,B). On the other hand, an inhibitory effect of about 75% was also observed after 30 min of incubation with oligomycin in both strains (Figure 3A,B), supporting the hypothesis that its lack of inhibition, shown in Figure 2, could depend on its difficulty of entry into the bacterial cell. This would explain why oligomycin has rarely been used as a control in experiments concerning ATP synthetic activity in bacteria.

### 2.4. Treatment with Cirsiliol, Quercetin, and Oligomycin Reduces the Endogenous ATP Content in a Time-Dependent Manner

**Figure 4:**
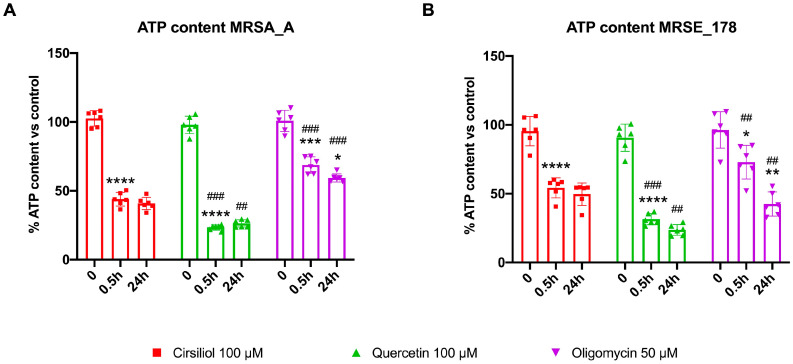
**Cirsiliol, quercetin, and oligomycin effects on ATP intracellular content in MRSA strain A and MRSE strain 178 after 0, 0.5, and 24 h of drug treatment**. (**A**) Percentage of ATP content in MRSA strain A (MRSA_A) following treatment with cirsiliol (100 μM), quercetin (100 μM), and oligomycin (50 μM) after 0, 0.5, and 24 h of drug treatment. (**B**) Percentage of ATP content in MRSE strain 178 (MRSE_178) following treatment with cirsiliol (100 μM), quercetin (100 μM), and oligomycin (50 μM) after 0, 0.5, and 24 h of drug treatment. In each panel, data are presented as mean ± SD and are representative of six independent experiments. The difference significance was tested by a one-way ANOVA test followed by the Tuckey’s test. *, **, ***, and **** indicate *p* < 0.05, 0.01, 0.001, or 0.0001 between 0 and 0.5 h or between 0.5 and 24 h since drug incubation. ## and ### indicate *p* < 0.01, or 0.001 between cirsiliol and quercetin or oligomycin treatments.

The ATP total concentration in MRSA_A and MRSE_178 was assessed at 0, 0.5, and 24 h after treatment with the drugs to investigate whether the inhibitory effect of cirsiliol, quercetin, and oligomycin on ATP synthesis exerted any consequences on the intracellular energy balance over time. Figure 4 shows that cirsiliol and quercetin caused a drastic drop in the intracellular ATP content as early as 0.5 h after incubation, with the values remaining low even 24 h after treatment for both strains (Figure 4A,B). Oligomycin caused a slight decrease in the ATP concentration after 0.5 h of incubation, in line with the hypothesis that its effect is slower due to a lower cell permeability, reaching lower levels after 24 h (Figure 4A,B). However, the oligomycin-dependent decrement in ATP levels was lower than that caused by cirsiliol and quercetin (Figure 4A,B). Preliminary observations also suggest that, similarly to quercetin, cirsiliol can affect the biofilm production in the MRSA and MRSE strains.

## 3. Discussion

This study evaluated the effect of two flavones, quercetin, and cirsiliol, on ATP synthesis and energy balance in clinical Gram-positive bacteria MRSA and MRSE strains, comparing the results to those obtained using oligomycin, the specific inhibitor of ATP synthase. Antimicrobial resistance (AMR) is one of the principal threats to global health, as declared in 2020 by the WHO (https://www.who.int/news-room/fact-sheets/detail/antibiotic-resistance (accessed on 3 July 2023)) [42]. According to the Review on Antibiotic Resistance updated in 2016, the number of deaths due to multi-drug resistance is around 7 × 10^5^ people/year and will cause an estimated cost of 100 trillion by 2050 [43]. Thus, non-antibiotic opportunities to treat bacterial infections have been proposed as possible options [26]. In this work, we chose two antibiotic-resistant strains of bacteria: MRSA and MRSE. MRSA remains an important pathogen in the EU area, despite a decline in MRSA isolation in 2011–2014 and subsequently in 2019 (https://www.ecdc.europa.eu/en/search?s=Antimicrobial+resistance+surveillance+in+Europe (accessed on 3 July 2023)). The MRSA spread depends on virulence factors that *S. aureus* can acquire in vivo through horizontal plasmid gene transfer in combination with β-lactam resistance [44]. In addition, MRSE owes its pathogenicity to its ability to produce a biofilm [45]. Biofilm formation is under the control of the quorum-sensing (QS) system [45]. QS relies on the release of chemical signals by bacteria (autoinducers), whose concentration rises as a function of cell density: the detection of a threshold concentration of the autoinducer leads to an alteration in gene expression. MRSE is of high clinical relevance as the employment of catheters, heart valves, and orthopedic prostheses causes many infections associated with it [46].

The first part of this study was devoted to optimizing the method for permeabilizing Gram-positive bacteria to assess ATP synthesis in real time. In detail, we modified the assay developed by Hari and Mori to assay ATP production in *E. coli* [37], inducing the permeabilization of the bacterial wall by low doses of Triton x-100 and a small osmotic shock attained by adding 20% of mQ water to the final volume of the assay (see Material and Methods section). To the best of our knowledge, this is the first study to quantitatively assay the dynamic ATP synthetic activity of Gram-positive MRSA and MRSE. The results showed that the ATP synthesis activity of MRSA and MRSE was approximately 2 and 6 nmol ATP/min/mg of total protein, respectively, suggesting that MRSE utilizes the aerobic pathway more than MRSA to produce energy. This difference could be consistent with the lower fitness of the former and the preference for an aerobic metabolism of the latter. In addition, the ATP synthesis trend, as far as could be detected by the assay, was linear over time. Concerning the effect of the flavonoid treatment, the results showed that cirsiliol inhibited ATP synthase in MRSA and MRSE instantaneously in a dose-dependent manner and that the reduction seen at the highest dose (100 µM) was slightly lower than that observed with quercetin used at the same concentration. Increasing the exposure time to the drugs led to an inhibition of around 75%, which had a long-term effect on the available ATP pool, suggesting that flavones alter bacteria’s bioenergetic state. On the other hand, literature data proposed that the primary antibacterial mechanism of flavonoids consists in energy metabolism inhibition [13]. In addition, crystallography studies showed that quercetin binds the ATP synthase F_1_ moiety, inhibiting its rotation [28], and our previous molecular docking analysis identified specific sites of interaction between cirsiliol and specific sites in ATP synthase [21]. The higher inhibiting effect of quercetin compared to cirsiliol could be explained considering the molecule chemical structure, as cirsiliol possesses two methoxy group substituents that render it a little more hydrophobic than quercetin, resulting in a minor cell wall permeability [47]. By contrast, oligomycin, a specific inhibitor of the F_o_ moiety of ATP synthase [41], did not demonstrate an immediate effect on the bacterial ATP synthetic activity but required at least a 30 min incubation to exert its action. On the other hand, the inability of 10 µM oligomycin to inhibit *S. aureus* ATP synthase was reported by two studies [48,49]. However, the same Authors showed that oligomycin was able to restore the sensitivity of intrinsically polymyxin-resistant MRSA to polymyxin B at 0.25× the minimum inhibitory concentration (MIC) [48].

Interestingly, the inhibition of ATP synthesis in *S. aureus* by polyphenols shows potential therapeutic effects, although ATP synthase is not essential for bacterial viability, as *S. aureus* is a facultative anaerobic bacterium [50]. For example, ATP synthase inhibition caused by resveratrol sensitized *S. aureus* to some human antimicrobial peptides to the same extent as the induction of the ATP synthase gene mutation *atpA* [51]. Similarly, the inactivation of genes coding for ATP synthase subunits increased the susceptibility to gentamicin [52], and mutations in genes coding for ATP synthase components inhibited the growth of *S. aureus* [53]. Tomatidine, a compound targeting ATP synthase, also selectively inhibited the growth of *S. aureus* variants with an inactivated electron transport chain (ETC-SCVs) [54], suggesting that ATP synthase inhibition also acts on the proton motive force produced by *S. aureus* under anaerobic conditions and necessary for the maintenance of its homeostasis. The impairment of the aerobic metabolism due to resveratrol treatment also plays a pivotal role in *P. aeruginosa* [55] and *E. coli* exposed to this molecule [56]. In addition, a combined proteomics and metabolomics study of the antibacterial properties of resveratrol reported that the inhibition of ATP synthesis is crucial for this action, in addition to other effects, including the decrease in the E1 subunit of pyruvate dehydrogenase and in succinate dehydrogenase and the inhibition of the TCA cycle and of oxidative phosphorylation [57]. The antimicrobial action of quercetin also includes a metabolic dysfunction [58]. Quercetin inhibited quorum sensing and biofilm production by *Vibrio parahaemolyticus*, inducing cell lysis [59], which was also observed in a strain of *S. epidermidis* (ATCC35984) [60]. It may be supposed that the ability of flavones, and polyphenols in general, to prevent biofilm formation and its resistance to antimicrobials implies an essential contribution of ATP synthase inhibition. Therefore, the present results and the literature data suggest that ATP synthase inhibition is a promising therapeutic strategy to sensitize MRSA and MRSE to therapeutic agents due to its ability to hinder the ATP supply, thus suppressing multiple ATP-dependent metabolic processes. On the other hand, it is well known that eukaryotic cell protection against oxidative stress takes advantage of polyphenols, which are components of a healthy diet. These compounds can modulate the functioning of the electron transfer chain, the main producer of free radicals, by interacting with ATP synthase. In this context, it may be supposed that the inhibition of ATP synthase by polyphenols and related molecules proved evolutionary advantageous for eukaryotic cells that possess a great capacity to produce chemical energy in the mitochondria, differently from bacterial cells, which are subject to a bioenergetic constraint [32].

## 4. Materials and Methods

### 4.1. Compounds

Cirsiliol (# SML0953), quercetin (# 1592409), and oligomycin (# O4876) were purchased from Sigma-Aldrich (St. Louis, MO, USA), belonging to the Merck’s group. Phosphate-Buffered Saline (PBS), Mueller Hinton broth and Columbia agar plates were purchased from Biolife Italiana S.r.l. (Milan, Italy).

### 4.2. Bacterial Strains, Culture, and Treatment

The MRSA_A and MRSE_178 strains were clinical isolates belonging to a collection obtained from the School of Medicine and Pharmacy of the University of Genoa (Italy), and identified by VITEK^®^ 2 (Biomerieux, Firenze, Italy). They were stored at −20 °C until their use, then they were streaked on Columbia agar plates and checked for their purity. In total, 5–6 colonies of each strain were inoculated in fresh Mueller–Hinton broth and incubated at 37 °C overnight. Subsequently, fresh broth was added to these cultures, which were re-incubated for 3 h. Appropriate sample aliquots were centrifuged and resuspended in PBS until a turbidity of 1 Mc Farland was obtained. The cultures were immediately used for subsequent experiments.

### 4.3. ATP Synthesis Evaluation in MRSA-A and MRSE_178

Due to the presence of a thick cell wall, bacteria must be permeabilized to allow the detection of ATP synthesis. For this scope, the method proposed by Hara and Mori was employed [37], with some modifications. All experiments started using a culture with a bacterial concentration of 5 × 10^7^ cfu/mL for both MRSA_A and MRSE_178. A glucose solution was added to this bacterial suspension to reach a final concentration of 10%.

To evaluate ATP synthesis, 1.25 μL of Triton X-100, 4 μL of DMSO, and 20 μL of mQ water were added to 30 μL of bacterial suspension to promote the bacterial wall permeabilization. ATP synthesis was assayed by the luciferin–luciferase method using the ATP Bioluminescence Assay Kit (Roche Diagnostics Corp., Indianapolis, IN, USA), adding to the mixture described above the same volume (50 μL) of a luciferin/luciferase solution. The reaction started with 0.1 mM final concentration of ADP. The drugs were used at the following final concentrations: 25 μM and 100 μM cirsiliol, 100 μM quercetin, and 50 μM oligomycin. In detail, the adjusted procedure involved incubating the bacterial suspension for 4 min with Triton X-100 (0.5% final concentration) and either DMSO (vehicle) or the same volume of each drug, then adding ultra-pure water (20%) and incubating the suspension for 4 more minutes before starting the assay by adding ADP and the luciferin/luciferase solution. The moderate osmotic shock, together with the detergent, proved necessary to allow hydrophilic molecules, such as adenine nucleotides, to permeate freely through the bacterial cell wall and be detectable by the assay kit. Notably, a longer exposure to the detergent causes the bacterial plasma membranes to uncouple, which leads to the inability to detect ATP synthesis by luminometry. 

In some experiments, the drugs were added immediately before ATP synthesis evaluation; in others, the drugs were added to the cultures 30 min before the ATP production assay. The intracellular total ATP content was assessed using the same luciferin–luciferase kit. In this case, the untreated or treated bacterial suspension was sonicated twice for 30 s with a 1 min pause between each cycle, on ice, to release the ATP contained in the bacteria.

ATP synthesis and ATP intracellular content were normalized against the sample protein concentration, which was evaluated by the Bradford method [61].

### 4.4. Statistical Analysis

Using the Prism 8 software, the data were analyzed using one-way ANOVA and then the Tukey’s multiple comparison test. The data are indicative of at least three independent experiments and are shown as mean standard deviation (SD). An error with probability *p* < 0.05 was considered statistically significant.

## 5. Conclusions

The increasing prevalence of untreatable infections poses a need for new antimicrobial agents. The ability of cirsiliol and quercetin to inhibit ATP synthesis in multi-resistant microorganisms, such as MRSA and MRSE, by interfering with their bioenergetics suggests that these flavonoids may have application in clinical practice. Cirsiliol and quercetin appear as promising candidates for enhancing the antibacterial activity of antibiotics, possibly reversing AMR. Cirsiliol and quercetin, being non-toxic to the human body, could be used as additives to enhance the antibacterial activity of antibiotics, since it would be impossible for any bacterium to develop resistance to ATP synthase inhibition. Furthermore, it is conceivable that these molecules inhibit ATP synthase rotation in both directions, i.e., the synthetic and the hydrolytic action of ATP, acting on both energy metabolism and the generation of the proton-motive force necessary for bacterial biology. Future studies may evaluate the possibility of using cirsiliol and quercetin against AMR.

## Data Availability

All data are contained within the article.

## Sample Availability

The compounds used in this work are commercially available and were purchased from Merck. In the Materials and Methods, the product codes are indicated.
